# Pre-collapse femoral head necrosis treated by hip abduction: a computational biomechanical analysis

**DOI:** 10.1007/s13755-022-00175-x

**Published:** 2022-05-14

**Authors:** Shaochi Li, Yan Liu, Guangquan Zhou, Wenjuan Zhang, Shengmei Wei, Jiajia He, Liao Shaoyi Stephen, Hang Wei

**Affiliations:** 1 Institute of Systems Engineering, Macau University of Science and Technology, Taipa, China; 2 Guangzhou Administration Institute, Guangzhou, China; 3 Dept of Information Systems, City University of Hong Kong, Kowloon Tong, Hong Kong; 4 The First Affiliated Hospital, Guangzhou University of Chinese Medicine, Guangzhou, China; 5 The First Affiliated Hospital of Kashi Prefecture, Kashi, China; 6 School of Medical Information Engineering, Guangzhou University of Chinese Medicine, Guangzhou, China

**Keywords:** Hip abduction, Stress transfer pattern, Load share ratio, Computational biomechanics, Parametric analysis, Computational models

## Abstract

**Background and objective:**

Clinical studies indicated that femoral head collapse (FHC) occurs in 90% of patients without intervention within five years after the diagnosis of femoral head necrosis (FHN). The management of the FHN is still a great challenging task. Clinical studies indicated that hip abduction as physical therapy represents an effective hip preservation method. However, the mechanism is unclear. In this study, we use computational biomechanical technology to investigate mechanical response in FHN patients with hip abduction and establish guide protocols for FHN rehabilitation.

**Materials and methods:**

Thirty computational models were constructed for evaluating the safety of hip abduction and comparing the biomechanical performance of hip abduction for the treatment of different necrotic classifications. The distribution of principal compressive stress (PCS) and load share ratio (LSR) were computed and used for biomechanical evaluation.

**Results:**

Before the start of physical therapy, when the size of necrotic segment is increased and located more laterally, the damage area of PCS enlarged and LSR of subchondral cortical to trabecular bone increased. As the increase of hip abduction angle, PCS of Type B transformed into Type A, PCS of Type C1 transformed into Type B, PCS of Type C2 transformed into Type C1; Except Type C2, the LSR return to normal level.

**Discussion and conclusion:**

Stress transfer damaged pattern correlated significantly with necrotic classification. Hip abduction motions effectively enlarge the area of PCS and recover the LSR of different structures by altering motion posture during gait. The results indicated that hip abduction may be an effective physical therapy in improving hip function and interrupt the disease pathway of FHC and THA.

## Introduction

Femoral head necrosis (FHN) is a relatively common disorder of the joint [1], which can damage the stress transfer path and break the load share balance of femoral head. Excessive non-physiologic mechanical changes in the hips with FHN cause subchondral cortical bone failure and the need for total hip arthroplasty (THA) [2]. Clinical studies indicated that femoral head collapse (FHC) occurs in 90% of patients without intervention within five years after the diagnosis of FHN [3]. The management of the FHN is still a great challenging task [4]. At present, THA has been considered as a standard and effective treatment option for patients with FHC in terms of pain relief and functional improvement; Hip preserving procedures as alternative treatments were developed to maintain or reconstruct the mechanical environment of the femoral head and interrupt the disease path of FHC and postpone the need of THA.

Clinical doctors and researchers share a common goal of choosing safe and effective hip preserving procedures for protecting the femoral head of the patients with pre-collapse FHN [5, 6]. These common procedures include core decompression, transtrochanetric rotation osteotomy (TRO), free and non-vascularized fibular grafting, fibular allograft. Isolated core decompression will accelerate a collapse and OA progression of the femoral head because of the lack of repaired materials and biomechanical structural support during the healing of the necrosis region [7–9]. Free vascularized fibular grafting can provide immediate structural support and vascularity, but it is often associated with serious trauma, technical difficulties and longer recovery time [10, 11]. TRO can improve the biomechanical properties in daily activity and effectively decreased the average stress by either anterior or posterior rotation [12]. However, it is a technically demanding procedure associated with high complication risks. It should be noted that these methods interrupting the disease path of FHC by altering the joint shape and/or structure were invasive treatment. They have involved restricted weight-bearing and bed rest after operations, which may result in osteoporosis, slow metabolism, muscle atrophy and poor clinical curative effect. Because of these unfavorable results, there has been considerable interest in evaluating treatment regimens that will reduce trauma and complications, improve the ability of motion in daily activity and prevent FHC.

Hip abduction as physical therapy represents an effective hip preservation method [13, 14], which can adjust stress transfer pattern of femoral head and load share ratio (LSR) of different structures by altering motion posture during gait. In theory, recovery of stress transfer function and load share relationship have beneficial disease-modifying effect on FHN. However, to our knowledge this regimen has not been previously evaluated for the treatment of FHN and it is unclear how to achieve these transformations. In this study, we use computational biomechanical technology to investigate mechanical response in FHN patients with hip abduction and establish guide protocols for FHN rehabilitation.

## Materials and methods

### JIC classification and research hypothesis

**Fig. 1:**
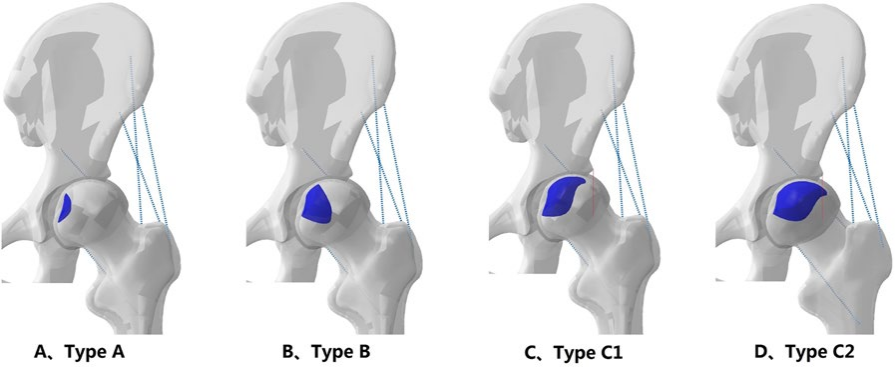
Three dimension model of JIC classification

In 2001, the Japanese Investigation Committee (JIC) revised diagnostic criteria to clarify the definition of osteonecrosis of the femoral head [15, 16]. According to the JIC classification criteria, FHN can be classified into subtypes A, B, C1 and C2, based on the location of the lesion in the weight-bearing area (as shown in Fig. 1). Type A lesions occupy the medial one-third or less of the weight-bearing portion, while Type B lesions occupy the medial two-thirds or less of the weight-bearing portion. Type C1 lesions occupy more than the medial two thirds of the weight-bearing portion without extending laterally to the acetabular edge. Type C2 lesions occupy more than the medial two-thirds of the weight-bearing portion and extend laterally to the acebtabular edge. We have postulated that hip abduction motion can improve the ability of motion in daily activity and prevent FHC for patients who diagnosis with pre-collapse FHN. To test this hypothesis, thirty parametric computational models were generated and the parametric definition of the models allowed study of the effect of abduction on biomechanical performance.

### Parametric modeling and boundary conditions

**Fig. 2:**
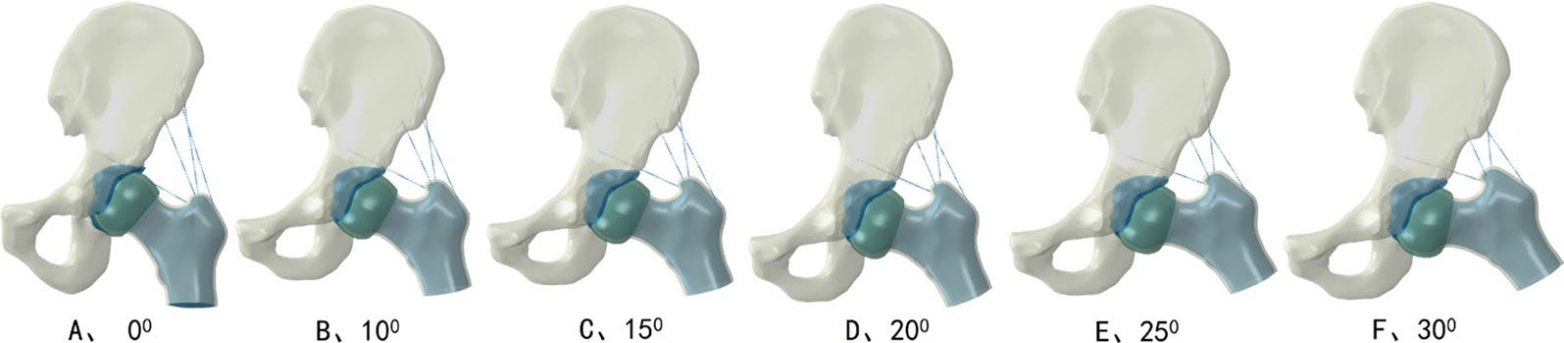
Hip abduction angle

Single-legged stance was considered a representative body position for the primary models. We used an abducent angleβ (β = 0°, 10°, 15°, 20°, 25°, 30°) along the anteroposterior axis of femur head to investigate the stress transfer pattern. The abducent variants were depicted schematically in Fig. 2. Finally, a total of 30 different computational models were used to simulate six hip abductions with five different progresses of FHN. The applied load was performed on a rigid plate that was tied to the distal femur. Constrains were applied on pubic symphysis and sacroiliac joint. Seven muscles were modeled as axial connectors muscle forces were depicted in literature [17]: adductor longus = 560 N; adductor magnus = 600 N; gluteal maximus = 550 N; gluteal medius = 700 N; gluteal minimus = 300 N; piriformis = 500 N; tensor fascia latae = 300 N.

## Results

### Distributions of principal compressive stress

**Fig. 3:**
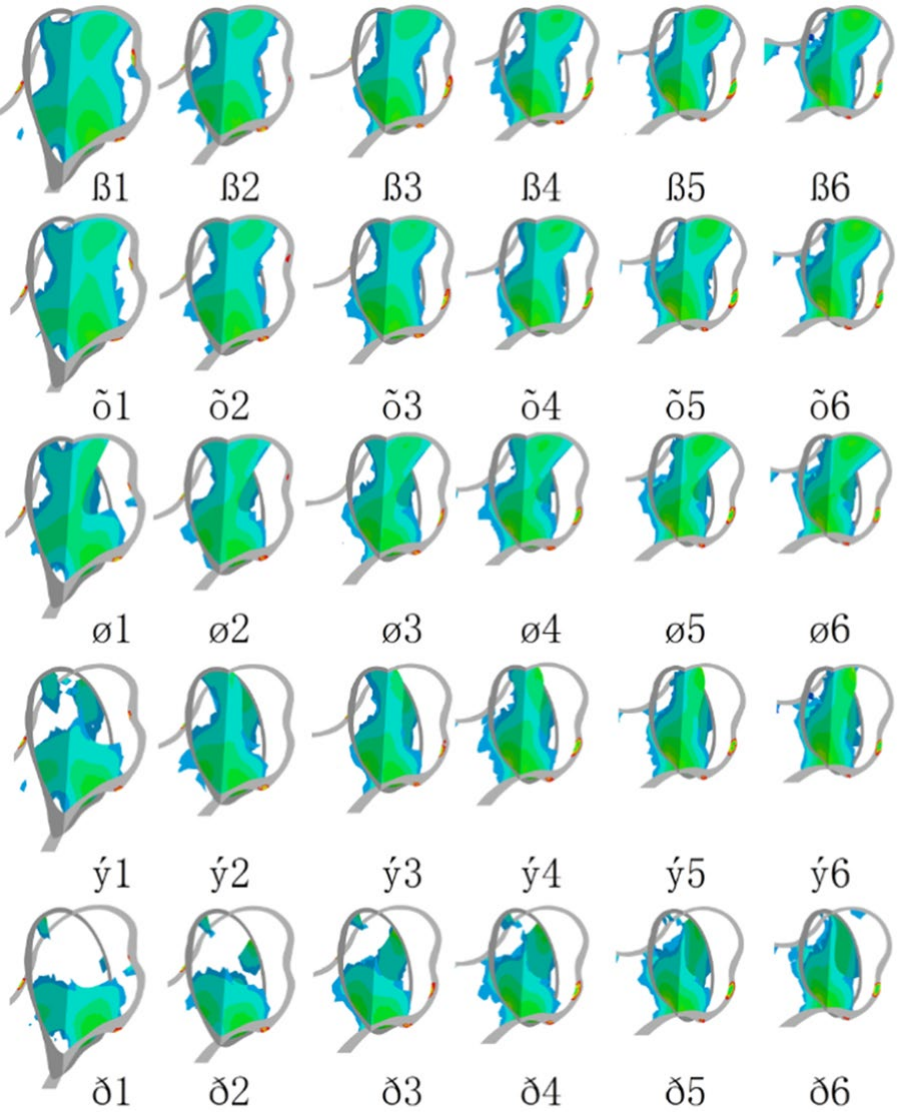
Distributions of principal compressive stress

The major function of principal compressive trabeculae in the femoral head is loading principal compressive stress (PCS) [18–20]. The distribution of PCS is an important index for evaluating the biomechanical performance of femoral head. In this work, hip abduction configurations of femoral head allow the study of PCS. Figure 3 showed the coronal-sagittal section of the distributions of PCT in femoral head. Figure 3ß1 shown that the PCS distributed along the principal compressive trabeculae in normal condition. When the size of the lesion segment is increased and located more laterally to the acebtabular edge (Fig. 3õ1 → ø1 → ý1 → ð1), the damage of PCS increasingly enlarge. Especially for Type C1 and C2, the PCS areas reduce by more than a half in both coronal and sagittal sections. After physical therapy, as shown in Fig. 3ß1–ß6, on the healthy condition the PCS located more laterally of the femoral head when the angle of hip abduction is increased. Figure 3õ2, ø2, ý2, ð2 → õ6, ø6, ý6, ð6 showed that the PCS area in coronal-sagittal section increased to varied extent. Especially for Type C1, the distribution of PCS only located within the femoral head below before physical therapy. When hip abduction angle is increased, the distribution of PCS was reconstructed from the top of the femoral head to the calcar.

### Efficiency of hip abduction

**Fig. 4:**
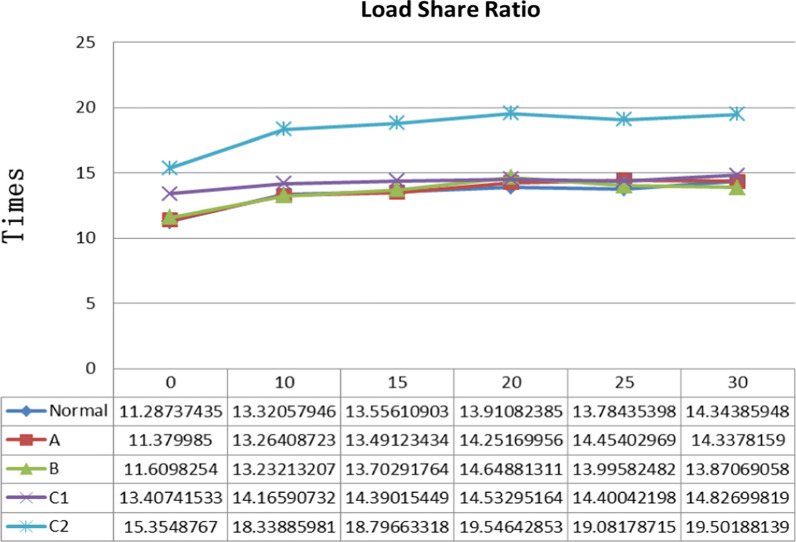
Load share ratios of femoral head

Average stresses are calculated from all the elements on the interesting region, which could reflect the efficacy of treatment option [12]. In this study, the average stresses of anterolateral column (S1) and principal stress transfer area (S2) were computed, respectively. Load share ratio (LSR) is an important index for evaluating the bearing-capacity of the femoral head, which is defined as the ratio of S1 to S2. Figure 4 displays the relationship between the LSR and abduction motion, and the difference among normal femoral head and four subtypes FHN. Before the start of physical therapy, there is no significant difference in LSR between the normal and necrotic femoral head with small lesion segment; when the size of necrotic segment is increased and located more laterally, LSR increase significantly. These indicate that large lesion segment (Type C1 and C2) breaks the load share relationship of different structure of femoral head. After physical therapy, LSR in Type C1 gradually transforms to normal level when hip abduction angles are more lager; while LSR in Type C2 still greater than the other subtypes and normal femoral head.

### Model validation

To validate the computational model at healthy conditions, the stress transfer pattern (Fig. 3ß1) vs. the morphological characteristics of the principal compressive trabeculae of cadaver bone and the bone density distributions in X-rays images were compared together [17, 18]. The simulation results are close to the previous studies results in the literature.

## Discussion

Hip joint is the largest weight-bearing structure of human, whose pathophysiology progression is closely related to the biomechanics. FHN is a relatively common rupture of joint homeostasis, which causes load share unbalance and the subchondral cortical bone buckle into the mechanical strength reduced area. The ideal treatment should aim at the recovery of the biomechanical performance in necrotic femoral head and interrupt the disease path of FHC and OA. The purpose of this study was to explore the effect of hip abduction motion on the biomechanical performance of pre-collapse FHN and establish guide protocols for physical therapy.

Previous studies indicated that physical therapy regimens have been useful for a variety of hip disorder [21–30]; only a few studies have investigated the effect of hip abduction on collapse risk of FHN. Lynne et al. [31] reported that a mean three-year hip survival rate of 82% in the necrotic femoral head treated with hip core decompression and physical therapy and 86% in those treated with physical therapy alone. Cui and Yuan [13] investigated the effect of Yuan’s Plastic therapy on treatment of FHN. They found a mean thirteen-month survival rate of 95.5% in the necrotic femoral head and this regimen can let patients with FHN carry weight earlier. Sun et al. [14] investigated the mechanism of biomechanics on the treatment of avascular necrosis of the femoral head with Anti-Chaplin gait by three-dimensional finite element method. The results indicated that Anti-Chaplin gait could reduce the load on the surface of femoral head and collapse risk of femoral head. The results indicated that the step width and abduction angle can affect stress distribution on the necrotic femur head. To our knowledge, this study is the first biomechanical study to investigate the PCS distributions and LSR in the femoral head for physical therapy of FHN.

Parametric modeling is an efficient and fast modeling strategy to manipulate the attribute of the bone in biomechanical analysis, which are suitable for creating a model family. In this study, an initial model of hip were generated based the anatomy of hip joint, and then four necrotic zone were generated by change the shape of necrotic geometry as soon as the dimension value is modified based on JIC. Six abducent angle were created as soon as the value is changed. In total, thirty computational models were generated and used to simulate a healthy and four necrotic femoral heads with six abduction angle. When β = 0°, our results show that as the size of the smaller necrotic segment becomes larger the likelihood of the rupture of arthrosis homeostasis increases as demonstrated by increasing LSR in subchondral bone and smaller PCS area in coronal-sagittal section. The stress transfer function in subtrondral trabeculae completely lose when the size of necrotic segment is more than the medial two thirds of the weight-bearing portion. Compared with the normal LSR, LSRs of four subtypes increase approximately 0.82%, 2.86%, 18.78% and 36.03%, respectively. According to load sharing theory [32] that if the absence of load share, hard tissue failure and nonunion rates greatly increase. When the LSR becomes larger, the likelihood of necrotic lesion healing may reduce and the risk of deterioration will increase. Previous studies have shown that Type C1/C2 FHN need surgical intervention because the risk of a collapse in the femoral head is quite high. While the results of this study provide a detailed description of the biomechanical evidence and physical therapy regimen to protect self-head of the patient with Type C1 FHN without invasive treatment. As the angle of hip abduction increases, the areas of PCS increase in both coronal and sagittal sections. PCS of Type B could be transformed into Type A, PCS of Type C1 could be transformed into Type B. PCS of Type C2 could be transformed into Type C1. The hip abduction motions also show that LSR of Type B and C1 reduce and return to the normal level when the β > 20°. While the LSR of Type C2 still increases approximately 35.96% more than the normal level, even though β = 30°. The results indicated that hip abduction regimen has a good biomechanical performance for the treatment of FHN and the biomechanical performance correlated significantly with necrotic classifications. It confirmed the safety of this physical treatment regimen for the patient with a necrotic lesion without extending laterally to the acebtabular edge and suggested that patients with FHN may carry weight earlier to improve biomechanical property without restricted weight-bearing and bed rest. The results also suggested that hip abduction combined with other treatment regimen on Type C2 FHN may get better clinical outcome.

## Conclusion

This study found that hip abduction motion added to the effectiveness of a physical therapy regimen for patients with pre-collapse FHN. The results of this study suggest that hip abduction motions apply to patients with a necrotic lesion without extending laterally to the acebtabular edge. This routine use of hip abduction motion combined with surgical treatment may get better clinical outcome in Type C2 FHN.
